# Supplementary material for: Bacterial Strains from Saline Environment Modulate the Expression of Saline Stress-Responsive Genes in Pepper (Capsicum annuum)
Source: Plants (Basel). 2023 Oct 15;12(20):3576. doi: 10.3390/plants12203576 (PMC10610202; doi:10.3390/plants12203576)
Supplement: Supplementary file 1 [file plants-12-03576-s001.zip › plants-2623273-supplementary.pdf]

Supplementary Materials

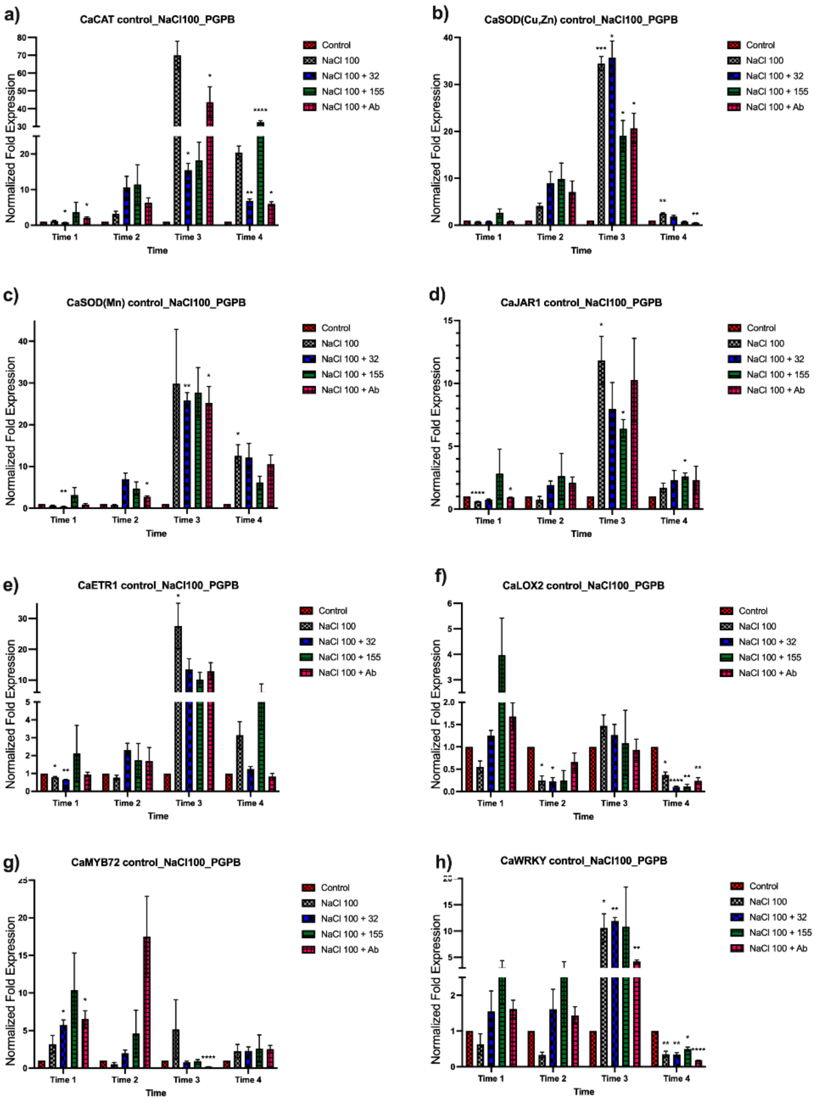

Figure S1. Gene expression analysis of stress-responsive genes of *C. annuum* plants inoculated with bacteria after 24 h of salt stress (100 mM NaCl). Quantitative RT-PCR determinations of relative expression levels of the genes: *CaCAT, CaSOD(Cu,Zn), CaSOD(Mn), CaJAR1*, *CaETR1*, *CaLOX2, CaMYB72*, and *CaWRKYa*. The data represented means of triplicate biological and experimental repeats; error bars represented SEM. The asterisk indicates statistically significant differences between treatments (range test P < 0.05, 0.01, and 0.001).

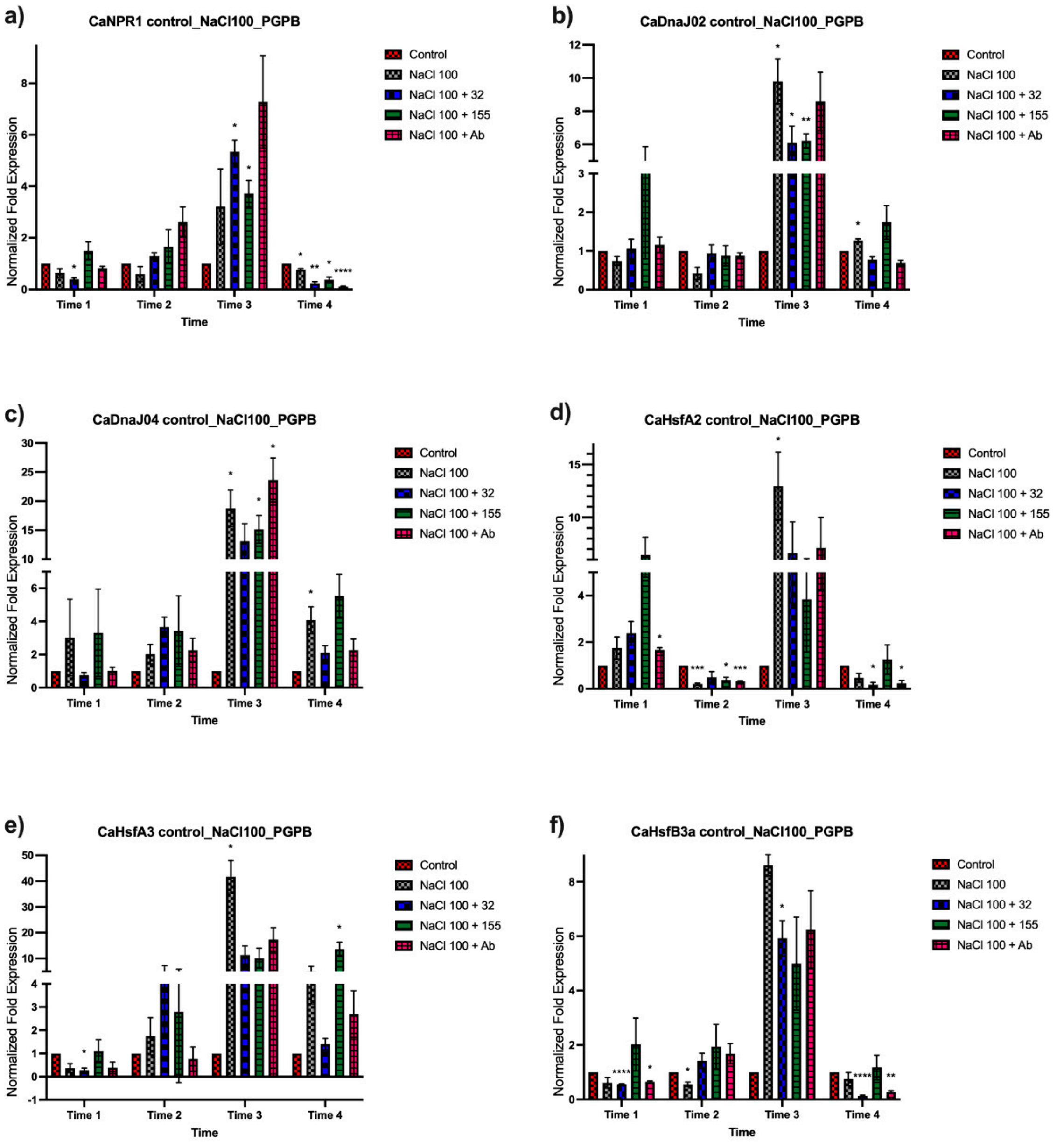

Figure S2. Gene expression analysis of NPR1, heat shock protein genes, and factors genes that regulation of genes that encode heat shock proteins of *C. annuum* plants inoculated with bacteria after 24 h of salt stress (100 mM NaCl). Quantitative RT-PCR determinations of relative expression levels of the genes: *CaNPR1*, CaDNaJ2, CaDNaJ04, *CahsfA*2, *CahsfA*3, and *CahsfB*3. The data represented means of triplicate biological and experimental repeats; error bars represented SEM. The asterisk indicates statistically significant differences between treatments (range test $P < 0.05$, 0.01, and 0.001).

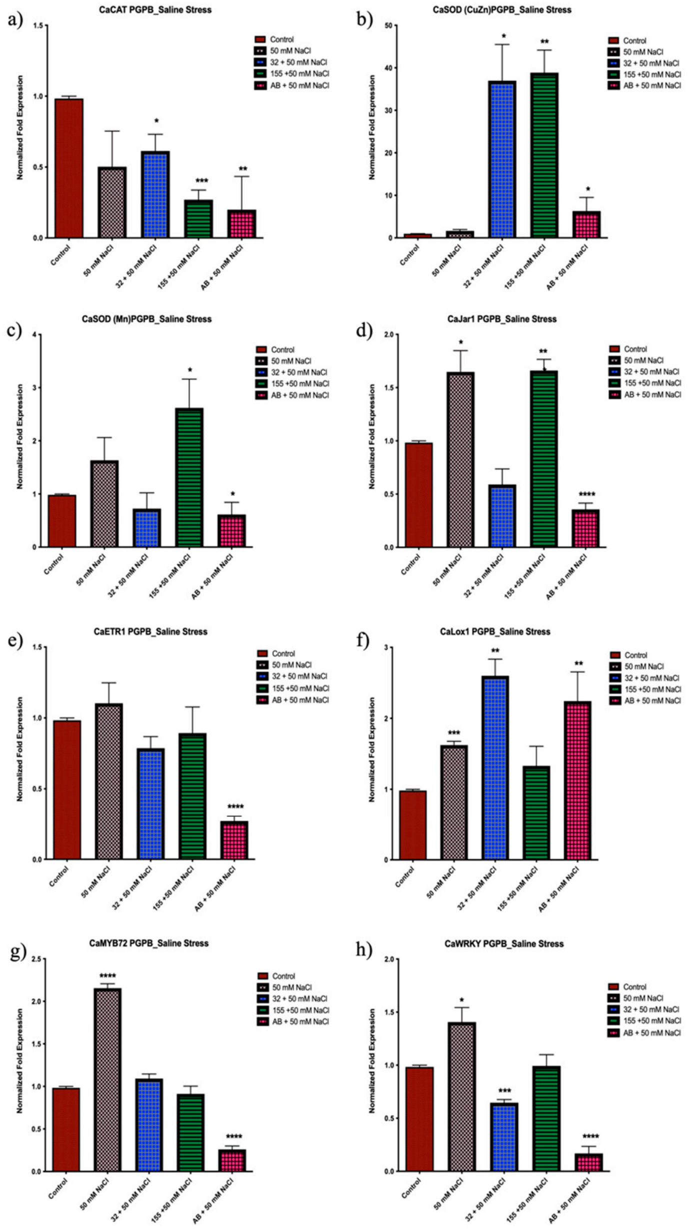

Figure S3. Gene expression analysis of stress-responsive genes of *Capsicum annuum* plants inoculated with salt stress (50 mM NaCl) after 24 h of bacteria inoculant. Quantitative RT-PCR determinations of relative expression levels of the genes: *CaCAT, CaSOD(Cu,Zn), CaSOD(Mn), CaJAR1*, *CaETR1*, *CaLOX2, CaMYB72*, and *CaWRKYa*. The data represented means of triplicate

biological and experimental repeats; error bars represented SEM. The asterisk indicates statistically significant differences between treatments (range test P < 0.05, 0.01, and 0.001).

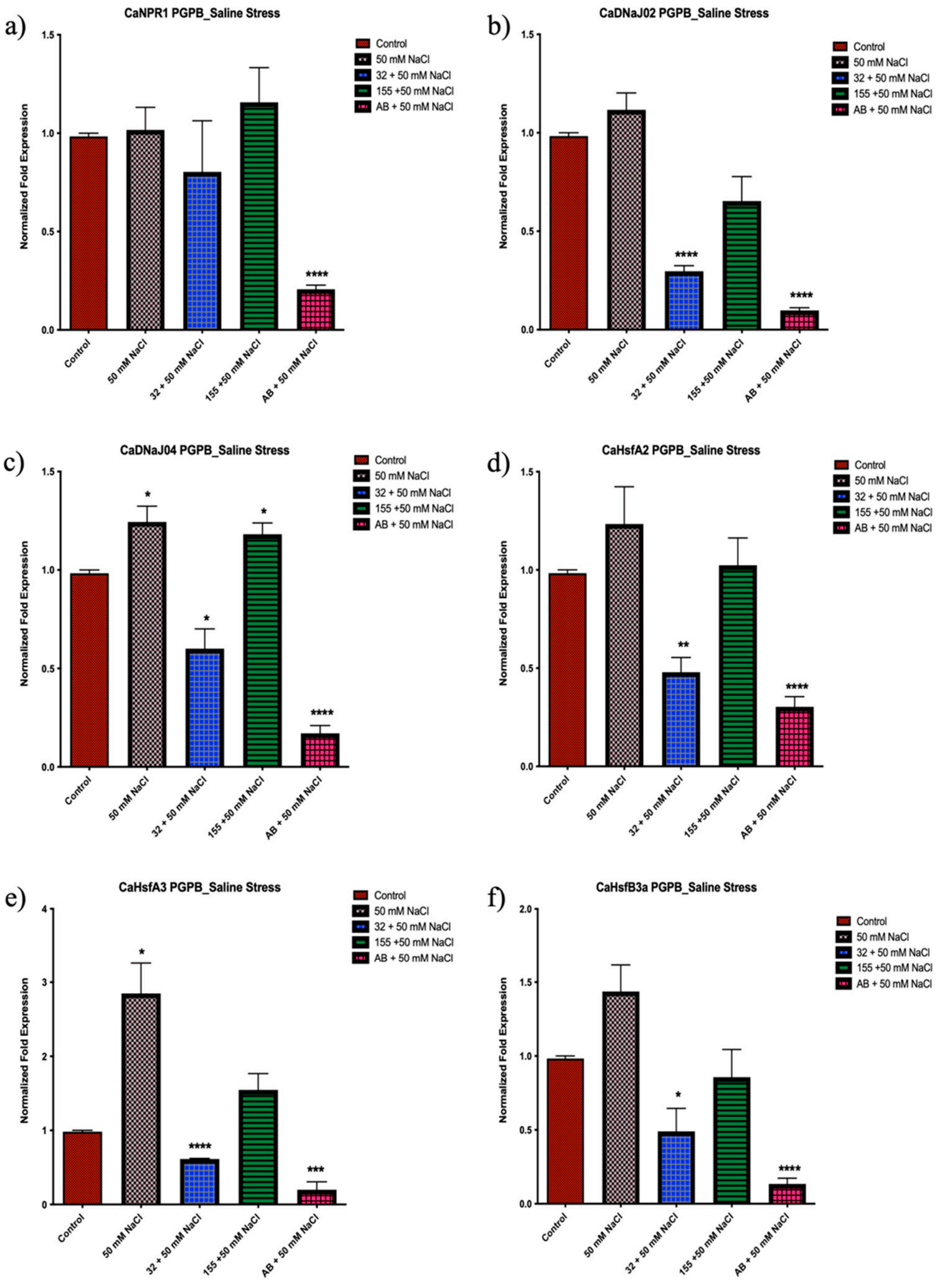

Figure S4. Gene expression analysis of NPR1, heat shock protein genes, and factors genes that regulation of genes that encode heat shock proteins of *C. annuum* plants inoculated with salt stress (50 mM NaCl) after 24 h of bacteria inoculant. Quantitative RT-PCR determinations of relative

expression levels of the genes: *CaNPR1*, CaDNaJ2, CaDNaJ04, *CahsfA*2, *CahsfA*3, and *CahsfB*3. The data represented means of triplicate biological and experimental repeats; error bars represented SEM. The asterisk indicates statistically significant differences between treatments (range test $P < 0.05$, 0.01, and 0.001).
